# Supplementary material for: Disentangling metabolic functions of bacteria in the honey bee gut
Source: PLoS Biol. 2017 Dec 12;15(12):e2003467. doi: 10.1371/journal.pbio.2003467 (PMC5726620; doi:10.1371/journal.pbio.2003467)
Supplement: S8 Data — (ZIP) [file pbio.2003467.s008.zip › Z-score_plots/86 microbial product 2,3,6-Trihydroxypyridine.pdf]

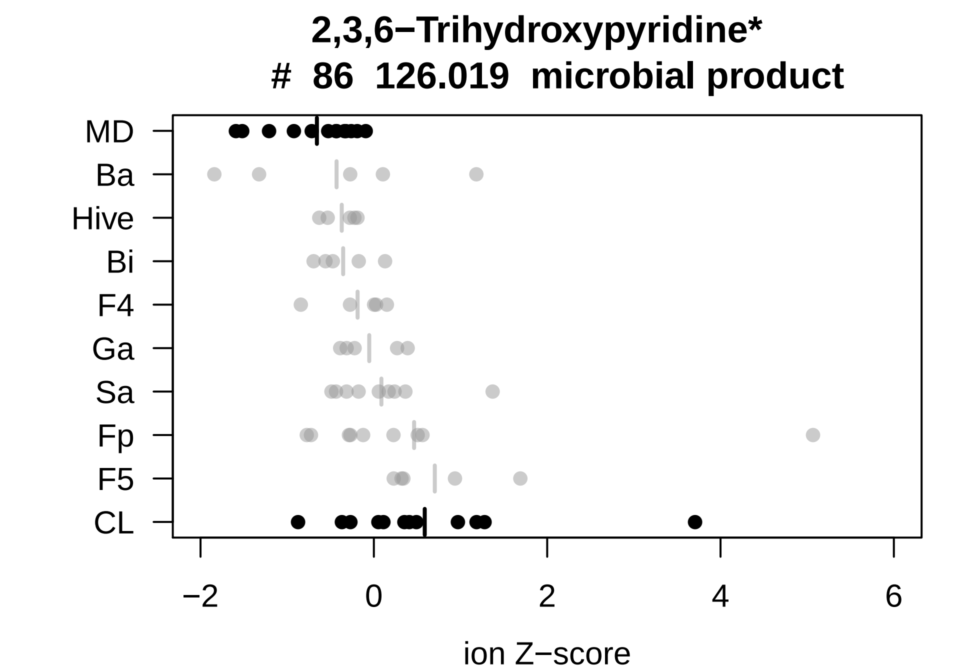

2,3,6−Trihydroxypyridine*
86 126.019 microbial product
MD
Ba
Hive
Bi
F4
Ga
Sa
Fp
F5
CL
−2
0
2
4
6
ion Z−score
